# Plan for Obtaining a More Perfect Fit of Vulcanite Plates

**Published:** 1867-12

**Authors:** W. Leigh Burton

**Affiliations:** Dentist, Richmond, Va.


### ARTICLE V.

### *Plan for obtaining a more perfect fit of Vulcanite Plates.*

By W. Leigh Burton, Dentist, Richmond, Va.

(Secretary of the Virginia Dental Society.)

It sometimes happens, after a vulcanite plate has been completed and tried in the mouth, that from some defect

---

*We have received from Dr. French a plaster model of the mouth which shows how successfully he has treated this case. *Editors.*

in the impression—particularly in very deep mouths—the plate does not fit the palate with sufficient accuracy to produce the necessary suction. If another impression is taken in plaster or wax and the plate made over, the chances are that the second attempt is apt to be a failure also. To insure certainty and save time, I have adopted the following simple plan :

Place a piece of soft wax on the posterior-palatine surface of the plate, or wherever the fault exists, place it in the mouth, press it with some force, and the pressure will cause the surplus wax to be forced out. Now take the plate and cover the palatine surface with plaster and allow it to harden. The space occupied by the wax is where the fault exists. The wax is then removed, and the plate, by being softened over a spirit lamp can be easily pressed down to its proper place.

